# Hospital financing of ischaemic stroke: determinants of funding and usefulness of DRG subcategories based on severity of illness

**DOI:** 10.1186/s12913-018-3134-6

**Published:** 2018-05-11

**Authors:** Sarah Dewilde, Lieven Annemans, Hilde Pincé, Vincent Thijs

**Affiliations:** 1 Department of Public Health, Faculty of Medicine, UGent, Gent, Belgium; 2 Services in Health Economics, Brussels, Belgium; 3 Interuniversity Centre for Health Economics Research UGent, VUB, Brussels, Belgium; 4 UZ Leuven, Leuven, Belgium; 5 KU Leuven Institute for Healthcare Policy, Leuven, Belgium; 6 Department of Neurology, Florey Institute of Neuroscience and Mental Health, University of Melbourne and Austin Health, Heidelberg, VIC Australia

**Keywords:** Ischaemic stroke, Costs, APR-DRG, SOI, Resource use, Funding

## Abstract

**Background:**

Several Western and Arab countries, as well as over 30 States in the US are using the “All-Patient Refined Diagnosis-Related Groups” (APR-DRGs) with four severity-of-illness (SOI) subcategories as a model for hospital funding. The aim of this study is to verify whether this is an adequate model for funding stroke hospital admissions, and to explore which risk factors and complications may influence the amount of funding.

**Methods:**

A bottom-up analysis of 2496 ischaemic stroke admissions in Belgium compares detailed in-hospital resource use (including length of stay, imaging, lab tests, visits and drugs) per SOI category and calculates total hospitalisation costs. A second analysis examines the relationship between the type and location of the index stroke, medical risk factors, patient characteristics, comorbidities and in-hospital complications on the one hand, and the funding level received by the hospital on the other hand. This dataset included 2513 hospitalisations reporting on 35,195 secondary diagnosis codes, all medically coded with the International Classification of Disease (ICD-9).

**Results:**

Total costs per admission increased by SOI (€3710–€16,735), with severe patients costing proportionally more in bed days (86%), and milder patients costing more in medical imaging (24%). In all resource categories (bed days, medications, visits and imaging and laboratory tests), the absolute utilisation rate was higher among severe patients, but also showed more variability.

SOI 1–2 was associated with vague, non-specific stroke-related ICD-9 codes as primary diagnosis (71–81% of hospitalisations). 24% hospitalisations had, in addition to the primary diagnosis, other stroke-related codes as secondary diagnoses. Presence of lung infections, intracranial bleeding, severe kidney disease, and do-not-resuscitate status were each associated with extreme SOI (*p* < 0.0001).

**Conclusions:**

APR-DRG with SOI subclassification is a useful funding model as it clusters stroke patients in homogenous groups in terms of resource use. The data on medical care utilisation can be used with unit costs from other countries with similar healthcare set-ups to 1) assess stroke-related hospital funding versus actual costs; 2) inform economic models on stroke prevention and treatment. The data on diagnosis codes can be used to 3) understand which factors influence hospital funding; 4) raise awareness about medical coding practices.

**Electronic supplementary material:**

The online version of this article (10.1186/s12913-018-3134-6) contains supplementary material, which is available to authorized users.

## Background

Stroke is one of the leading causes of morbidity and disability in adults [1], with total acute care costs in the first year after stroke varying between €5435 and €13,409 per patient [2–6], representing 2–4% of total health care expenditures [7]. In many countries these medical care costs are mainly funded by a national insurance system, creating the need for an efficacious and equitable funding system balancing the financial risks born by the provider and the National Health System with the health risk born by the patient. Many countries fund hospitals through derivatives from the Diagnosis Related Groups (DRG) [8], a classification system aiming to group homogenous diagnostic patients with equivalent use of hospital resources to calculate the “average” cost of a hospitalisation in order to assign the correct amount of funding to the health care provider [9].

Some, but not all, DRG systems provide a further division into subcategories for a better match between the funding received and the expenses made by hospitals for treating the patient [10]. In the All Patient Refined (APR)-DRG system, the basic DRG structure is expanded with 4 subclasses relating to severity of illness (SOI) [10]. This particular classification is applied as a model for reimbursement in over 30 different states in the US, and also in Belgium, Spain and Italy, and in some Arab countries [11]. The 4 SOI subclasses represent respectively, minor, moderate, major or extreme physiologic decompensation or loss of function of an organ system. Extreme SOI is usually associated with multiple comorbidities involving 2 or more organ systems [12], and is assigned to patients with poor outcomes in spite of receiving more frequent and intensive treatment. The distribution of hospital admissions across these subclasses within a DRG has a substantial impact on hospital budgets as well as on public health care expenditure on stroke. Gaining insight into the factors that determine the categorisation of stroke patients into subclasses of the DRG is therefore relevant for hospitals as well as public authorities.

This study first aims to document actual resources used in hospital by ischaemic stroke patients and to verify to which extent this varies according to SOI categories, in order to assess whether the division into DRG subcategories is indeed justified. The second objective is to explore which medical risk factors and complications are significantly related to higher SOI and thus higher funding.

## Methods

Two datasets were obtained in order to address the two aims of this study:

### Analysis of in-hospital resource use by SOI

To compare resource use by SOI, retrospective aggregate data on ischaemic stroke admissions classified as APR-DRG 045 “CVA and precerebral occlusion with infarct” from the administrative department of 5 Belgian hospitals were obtained for the years 2008 up to 2011, reporting on the total number of bed days, number and type of medical imaging and clinical biology tests, and drug expenditures for their admitted patients, by SOI. Participating hospitals were distributed across the country and included regional as well as teaching hospitals. Average bottom-up costs were calculated according to the recommended method by the Belgian Health Care Knowledge Centre [13, 14]. The unit costs applied to each resource item took into account three different financing sources received by hospitals for each in-patient admission: the fee-for-service [13, 14] received from the National Compulsory Insurance Fund, patient contribution per day or per admission [13, 14], and lump sum payments paid on a yearly basis by the National Institute for Health and Disability Insurance (NIHDI). The latter – lump sum payments – was calculated by applying a multiplication factor to the unit costs, derived by the ratio between the historical national average of total funding for a given resource category and the total fee-for-service payments. This factor was estimated to approximate 5.0 for laboratory tests and 1.7 for imaging [13]. Detailed data on specialists visits was not available, therefore the cost of these visits was approximated by the standard reimbursement tariff for routine visits [14]. This tariff is derived from a calculation based on the length of stay with degressive amounts paid for longer stays. The reference year for the costs was 2014. Total bottom-up costs were calculated per hospital admission and per SOI level and were compared to the top-down national reimbursement received by hospitals for a stroke admission. The national aggregate data on 14,911 ischaemic stroke admissions (APR-DRG 045) was obtained from the Belgian TCT database (2011) [15] and used for comparison with our sample. This national registry collects the medical diagnoses anonymously from all patients that are admitted overnight to all acute Belgian hospitals and the costs charged to the NIHDI for each admission.

### Analysis of primary and secondary diagnosis codes by SOI

All ischaemic stroke patients who were admitted between 2008 and 2014 to the largest teaching hospital in Belgium (UZ Leuven) were included in a second dataset. Ischaemic stroke hospitalisations were identified by filtering on the APR-DRG code “045 CVA & Precerebral Occlusion with Infarct”, with specification of a primary diagnosis code “433.x Occlusion and stenosis of precerebral arteries” or “434.x Occlusion of cerebral arteries”. These codes are based on the International Classification of Diseases, Version 9, Clinical Modification (ICD-9). In addition, the dataset included ICD-9 codes of the event leading to the hospitalisation, of pre-existing comorbidities, of in-hospital complications, of surgical or medical procedures, of short- and long term sequelae, and of age and gender of the patient, which are all labelled as secondary diagnoses. A proprietary algorithm created by 3M Health Information Systems (APR-DRG v15.0) combined these primary and secondary diagnoses codes to assign a DRG and SOI to each admission [12]. This coding algorithm is not published, and not known to the administrative department of the hospital responsible for the coding of the medical files. All diagnosis codes associated with higher SOI levels, and therefore higher reimbursement, were identified. A Poisson regression with log link compared the occurrence of diagnosis codes between the higher and the lower SOI levels, and in cases with low cell count Fisher’s exact testing was applied. All analyses were conducted in SAS. No funding was received for this study.

## Results

### Analysis of in-hospital resource use by SOI

In this bottom-up costing analysis, 2496 stroke admissions were included. Of these, all admissions accounted for length of stay (LOS) calculations, 2364 for medical imaging, and 1954 for medications whereas data for honoraria to specialists were not available. In total, the sample captured 4.3% of all ischaemic stroke hospital admissions in Belgium during that period [15]. The average costs incurred during stroke admission for LOS, specialist visits, medications, medical imaging and clinical biology tests for each SOI level are detailed in Table 1.

**Table 1:** Average hospitalisation cost of ischaemic stroke by SOI based on the retrospective dataset (bottom-up cost)

Retrospective aggregate data (2008–2011)	SOI 1	SOI 2	SOI 3	SOI 4	ALL
Number of stroke admissions	98	1292	659	447	2496
%	3.9%	51.8%	26.4%	17.9%	100%
Average LOS in days (SD)	6.06 (1.28)	10.81 (1.22)	19.74 (7.14)	35.23 (12.52)	17.34 (4.48)
LOS cost^a^	€2480	€4371	€7972	€14,352	€7032
Medications	€143.58	€156.96	€361.75	€780.25	€324.22
Visits^b^	€138.81	€178.36	€220.51	€284.96	€210.54
Clinical biology^c^	€64.54	€64.54	€64.54	€64.54	€64.54
Medical imaging^d^	€882.78	€872.49	€936.27	€1253.65	€963.01
Total cost per stroke admission	€3710	€5643	€9555	€16,735	€8594

SOI: severity of illness, SD: standard deviation; LOS: length of stay

^a^Claims data, average of €406.96 per day

^b^Approximated by reimbursement tariff: calculated as €26.00 for days 1–5; €8.33 for days 6–12 and €4.16 thereafter

^c^See calculations Additional file 1: Table S1

^d^See calculations Additional file 2: Table S2

The most frequently assigned subcategories were SOI 2 and SOI 3, representing 78.2% of all stroke admissions. The average LOS in an acute ward had a strong association with SOI and represented 67, 77, 83 and 86% of total costs in SOI 1 to 4, respectively; its variability also increased with higher SOI levels. Average spending on medications varied also by SOI, with drug costs in SOI 4 being 5.5 times higher compared to SOI 1, representing between 4 and 5% of total costs in all SOI classes. On average 15 types of laboratory tests were used including haematology tests, coagulation, renal and liver function tests, electrolytes, heart enzymes, glucose and lipid profiles as well as C-reactive protein (details in Additional file 1: Table S1). These resources did not differ by SOI in our dataset and were part of a general work-up at admission (0.4–1.7% of total costs). A total of 39 different medical imaging and other tests were used during stroke hospitalisations, including functional measurements, Holter tests, angiography, arteriography, echography, duplex ultrasonography, electromyography, electroencephalography, radiology, x-rays, computed tomography and magnetic resonance imaging. The rate of use of each test by SOI is displayed in Additional file 2: Table S2, and also increases by SOI: higher SOI is associated with a higher number of diagnostic imaging tests and higher associated costs. This cost component, though increasing with SOI, represented a smaller proportion of total costs in more severe subclasses (24, 15, 10 and 7% of total costs in SOI 1 to 4, respectively). Total costs were found to be significantly associated with SOI, with the most severe stroke (SOI 4) being 4.5 times more expensive than a mild case (SOI 1).

Table 2 displays the national average health care expenditures for ischaemic stroke patients in 2011 [15]. The distribution of stroke admissions across the different SOI levels in our bottom-up analysis was not dissimilar to the national distribution, with mild hospitalisations being slightly under-represented in favour of severe hospitalisations (Table 1 compared with Table 2). The average LOS from our bottom-up approach was lower as compared to the national top-down approach (17.3 vs 18.6 days). Summing up the categories specialist visits, medical imaging and clinical biology, average cost per stroke admission was approximately €1238 per patient in our analysis compared to the national average category of “honoraria” of €1909 per patient. This honoraria category includes in addition to the types of resources incorporated in our study (specialist visits, medical imaging and clinical biology) also medical procedures, medical night supervision, implants and prostheses, and a rest category covering among others incontinence material, blood, radio isotopes, implants and others. These resources were not recorded in our bottom-up approach. Total cost per stroke admission in the bottom-up analysis (€8594) was about 5% lower compared to the national average (€8987). This is more pronounced in SOI 2 and SOI 3, where the total bottom-up costs are respectively 12.4 and 12.8% lower compared to the national average, and less pronounced in SOI 4 (− 1.9%) and SOI 1 (+ 2.6%).

**Table 2:** National average hospitalisation cost of ischaemic stroke by SOI (top-down cost)

National aggregate data (2011)	SOI 1	SOI 2	SOI 3	SOI 4	ALL
Number of stroke admissions	785	7910	4235	1981	14,911
%	5.3%	53.0%	28.4%	13.3%	100%
Average LOS (days)	6.6	13.1	23.7	34.4	18.6
LOS cost	€2452.93	€4779.79	€8472.71	€12,910.79	€6786.39
Medications	€71.69	€151.05	€307.12	€903.69	€291.19
Visits, clinical biology and medical imaging	€1091.64	€1510.84	€2182.44	€3238.36	€1909.03
Total cost per stroke admission	€3616	€6442	€10,962	€17,053	€8987

SOI: severity of illness, LOS: length of stay

Source: Belgian TCT database, 2011^(15)^

### Analysis of primary and secondary diagnosis codes by SOI

#### Primary diagnosis codes (location and type of the ischaemic stroke)

A total of 2513 stroke admissions were recorded during the study period 2008–2014, distributed across SOI classes as follows: 52 (2.0%) in SOI 1; 1233 (49.1%) in SOI 2; 797 (31.7%) in SOI 3; and 431 (17.2%) in SOI 4. Each admission was associated with a single primary diagnosis related to the reason for admission, and one or more secondary diagnoses related to comorbidities, complications, procedures, and patient characteristics.

The distribution of patients across each stroke-related primary diagnosis code is displayed in Table 3. One of the primary ICD-9 diagnosis codes related to the cerebral arteries was non-specific on the type of the event (thrombosis or embolism) and non-specific on the location in the brain (“Unspecified occlusion of the cerebral artery, with mention of brain infarct” ICD-9434.91), whereas other ICD-9 codes were differentiated into thrombosis, embolism, or occlusion and stenosis; or were more precise on the location of the stroke, which may influence the long-term deficits patients can experience. On average, 64.5% of ischaemic strokes were attributed to this non-specific category (Table 3), but this was significantly more frequent in lower SOI categories (*p* < 0.0001), with up to 81 and 71% of hospitalisations in SOI 1 and 2 respectively being assigned this code as primary diagnosis. The other primary diagnosis codes were more specific on either the type of event or the location of the stroke, but no relationship was found between each individual stroke code and higher SOI.

**Table 3:** Primary diagnosis codes associated with APR-DRG 045 ischaemic stroke

Description of primary diagnosis codes within APR-DRG 045	% of total	Distribution per SOI			
		SOI 1	SOI 2	SOI 3	SOI 4
N	2513	52	1233	797	431
%	100.0	2.0	49.1	31.7	17.2
Unspecified occlusion of the cerebral artery, with mention of brain infarct (ICD 434.91)^a^	64.5%	80.8%	71.0%	58.9%	54.5%
Cerebral embolism, with mention of brain infarct (ICD 434.11)	21.9%	7.7%	17.6%	28.1%	24.4%
Occlusion and stenosis of the carotid artery, with mention of brain infarct (ICD 433.11)	6.2%	7.7%	5.0%	6.2%	10.0%
Cerebral thrombosis, with mention of brain infarct (ICD 434.01)	2.8%	1.9%	1.8%	2.8%	5.8%
Multiple and bilateral occlusion and stenosis of the precerebral arteries, with brain infarct (ICD 433.31)	1.8%	1.9%	1.5%	1.6%	2.6%
Occlusion and stenosis of the basilar artery, with mention of brain infarct (ICD 433.01)	1.6%	0.0%	1.5%	1.5%	2.3%
Occlusion and stenosis of the vertebral artery, with mention of brain infarct (ICD 433.21)	1.2%	0.0%	1.6%	0.9%	0.5%
Occlusion and stenosis of other specified precerebral arteries, with brain infarct (ICD 433.81)	0.1%	0.0%	0.1%	0.1%	0.0%

ICD: International Classification of Diseases, SOI: severity of illness

#### Secondary diagnosis codes (comorbidities and complications)

A total of 35,195 secondary codes were retrieved from the dataset representing 2241 unique ICD-9 codes. As expected from the SOI definition, we found a significant positive relationship between the average number of secondary diagnoses per admission and the SOI: on average 4.1 secondary diagnoses were assigned per ischaemic stroke admission in SOI 1, 9.9 in SOI 2, 16.5 in SOI 3 and 22.4 in SOI 4, clearly indicating that the involvement of multiple organ systems leads to higher severity levels.

Typical risk factors in ischaemic stroke patients include previous myocardial infarction, diabetes, atrial fibrillation, hypertension, hypercholesterolaemia, smoking and atherosclerosis. Most of these risk factors were found not to be individually linked to more severe SOI levels, as they were present across all SOI levels (Table 4), apart from atrial fibrillation and diabetes. Even though the sample size was small in SOI 1, none of these patients had atrial fibrillation or diabetes. Atrial fibrillation is a comorbidity typically occurring in up to 30% of stroke patients [16, 17], and therefore should presumably have been found in about 15 patients in our SOI 1 sample. Similarly, the prevalence of diabetes in stroke patients ranges from 13.2 to 36.9% which corresponds with 7 to 19 of the 52 patients in our SOI 1 sample [17–19]. Statistical testing with Fisher’s exact test confirmed that this was not a sampling problem but instead that these two risk factors are structurally linked to a SOI level of at least 2. The other typical stroke comorbidities such as previous myocardial infarction, hypertension, hypercholesterolaemia, smoking and atherosclerosis do not individually lead to the attribution of a higher SOI, but the combination of these comorbidities however will generate a higher SOI assigned to the hospitalisation. The most commonly observed secondary codes in ischaemic stroke patients were hypertension (60%), hypercholesterolaemia (60%), hemiplegia, monoplegia or paraplegia (59%), speech disorder (56%), apraxia, dysphagia, ataxia, vertigo or cognitive dysfunction (35%) (Table 4).

**Table 4:** Secondary codes by SOI occurring in at least 10% of all ischaemic stroke patients

Description of secondary diagnosis code	SOI 1	SOI 2	SOI 3	SOI 4	ALL
Number of stroke admissions	52	1233	797	431	2513
Typical stroke risk factors					
Atherosclerosis	9.6%	17.7%	21.5%	20.0%	19.1%
Arrhythmia and other dysrhythmia	7.7%	14.1%	18.1%	18.3%	16.0%
Atrial fibrillation	0.0%	18.4%	45.9%	45.5%	31.4%
Diabetes	0.0%	19.1%	26.7%	29.2%	22.8%
Obesity, overweight	1.9%	16.4%	17.6%	10.0%	15.4%
Hypertension	40.4%	62.4%	58.2%	55.9%	59.5%
Hypercholesterolaemia/ hyperlipidaemia	21.2%	67.8%	56.5%	49.2%	60.0%
Smoking	23.1%	20.6%	10.9%	10.4%	15.8%
Previous myocardial infarction	1.9%	8.2%	12.8%	16.9%	11.0%
Previous stroke	7.7%	14.6%	16.6%	16.0%	15.3%
Other secondary diagnosis codes^a^					
Hemiplegia, paraplegia, monoplegia^b^	7.7%	50.1%	65.6%	78.2%	59.0%
Speech disorder^b^	17.3%	48.8%	60.0%	71.5%	55.6%
Apraxia, dysphagia, ataxia, vertigo, cognitive dysfunction	19.2%	29.0%	34.1%	53.6%	34.6%
Facial paralysis^b^	9.6%	29.8%	35.6%	42.5%	33.4%
Mental illness^c^	1.9%	12.4%	29.5%	36.4%	21.7%
Long term use of anti-coagulation	1.9%	14.9%	26.0%	23.4%	19.6%
Loss of half of the visual field in both eyes	0.0%	9.7%	27.4%	27.6%	18.2%
Living alone	7.7%	10.2%	17.9%	23.7%	14.9%
Mitral and/or aorta valve stenosis or insufficiency	3.8%	8.5%	17.4%	20.9%	13.4%
Atrial septal defect	0.0%	14.7%	10.7%	5.6%	11.5%
Hypertensive Chronic Renal disease	0.0%	5.9%	15.1%	17.9%	10.7%
Cancer: neoplasm, lymphoma, carcinoma, myeloma, metastases	1.9%	6.6%	12.0%	16.0%	9.8%
Stroke-related secondary diagnosis					
Any stroke-related diagnosis ICD-9 code^d^	11.5%	21.2%	24.5%	32.7%	24.0%

ICD: International Classification of Diseases, SOI: severity of illness

^a^Diagnosis codes including: pre-existing co-morbidities, in-hospital complications, patient characteristics, medical care

^b^Neurological deficits due to stroke

^c^Including dementia, delirium, Alzheimer, delirium, cognitive dysfunction

^d^Including all 433.x and 434.x ICD-9 codes and subcategories

The high number of hospital admissions that have another stroke-related ICD-9 code as one of the secondary codes is noteworthy. About 24% (607/2513) of patients had one or more secondary stroke diagnosis codes in their electronic DRG file.

Table 5 displays a list of secondary codes occurring significantly more in SOI 3 and SOI 4 as compared to lower SOI levels. These represent comorbidities that mostly occur in the highest severity levels and are indicative of multiple organ systems affected and higher resource utilisation. The comorbidities included, among others, pneumonitis, pneumonia, intracranial haemorrhage, renal insufficiency and do-not-resuscitate status for SOI 4; and neurological complications, anaemia, Urinary Tract Infection (UTI), hypokalaemia and heart failure or heart decompensation for SOI 3 and 4. These codes will lead to the attribution of a higher severity level, and thus higher funding for hospitals.

**Table 5:** Secondary diagnosis codes occurring more frequently in SOI 3 and SOI 4

Occurring significantly more frequently in SOI 4	SOI 1 *N* = 52	SOI 2 *N* = 1233	SOI 3 *N* = 797	SOI 4 *N* = 431	*p*-value^b^
Pneumonia	0%	0%	6%	30%	< 0.0001
Pneumonitis	0%	0%	4%	28%	< 0.0001
Palliative care	2%	3%	7%	20%	< 0.0001
DNR status	0%	1%	7%	16%	< 0.0001
Intracranial haemorrhage	0%	0%	6%	15%	< 0.0001
Feeding problems, malnourishment	0%	0%	1%	12%	< 0.0001
Renal insufficiency (acute and chronic)	0%	0%	2%	7%	< 0.0001
Occurring significantly more frequently in SOI 3 and SOI 4	SOI 1 *N* = 52	SOI 2 *N* = 1233	SOI 3 *N* = 797	SOI 4 *N* = 431	p-value^c^
Infection	2%	2%	33%	65%	< 0.0001
Neurologic complications^a^	0%	1%	15%	40%	< 0.0001
Urinary tract infection	0%	1%	22%	29%	< 0.0001
Anaemia	0%	5%	13%	27%	< 0.0001
Rehabilitation	0%	7%	17%	25%	< 0.0001
Heart failure and heart decompensation	0%	0%	15%	23%	< 0.0001
Hypokalaemia	0%	3%	14%	20%	< 0.0001
Pulmonary heart disease	0%	0%	5%	8%	< 0.0001
Oesophagitis	0%	1%	3%	8%	< 0.0001

SOI: severity of illness, DNR: do not resuscitate

^a^Unconsciousness, coma, cerebral compression, cerebral oedema

^b^p-value for Likelihood Ratio test type 3 from a Poisson Log model: SOI 4 versus SOI 1, SOI 2 and SOI 3 combined

^c^*p*-value for Likelihood Ratio test type 3 from a Poisson Log model: SOI 3–4 combined versus SOI 1–2 combined

## Discussion

### SOI subclassification of DRG to fund ischaemic stroke admissions is meaningful

Our first analysis on in-hospital resource utilisation compared the types (bed days, medical imaging, medications, specialist visits, and clinical laboratory tests) and the amount of medical resources used per patient at each SOI level, and showed that in absolute terms as well as proportionally these varied substantially between SOI categories. More severe patients had proportionally more bed day costs and fewer imaging costs than milder patients, however they consumed a higher amount of resources across all categories. These findings indicate that ischaemic stroke patients are heterogeneous in their medical care consumption, and a split of the DRG in SOI subcategories to obtain groups of patients who are more homogenous in their resource use, is justified for ischaemic stroke admissions. In addition, we observed that the mix of resources used at different SOI levels also lead to differences in total costs, therefore subcategorisations into SOI will lead to more efficient funding within each category. When hospitals have the same patient distribution across SOI as in the national dataset, funding of ischaemic stroke hospitalisation with a single DRG code is adequate. But when hospitals admit proportionally more patients with higher severity of illness, they will be underfunded when receiving only a uniform payment per patient.

Our dataset spanned 4 years’ time and within this time, no major shifts were recorded in terms of resources used per SOI. The detailed documentation of these resources can be used with unit costs from jurisdictions with similar health care set-ups to estimate in-hospital costs. These costs can be used in economic models and health-care evaluations to estimate costs of stroke prevention or acute stroke care, or to better allocate health care resources. Many states in the US fund their hospitals in part through the APR-DRG-SOI algorithm. In contrast to many European countries, the care given to patients in the US may differ whether they fall under Medicaid (19% of the population), Medicare (14%), an employer-subsidized health plan (49%), a privately paid health plan (7%), or are uninsured (11%) [20]. It may be possible that the medical resource utilization we calculated by SOI, differs within each SOI category by the insurance status of the patient. There is evidence among stroke patients that insured patients [21] arrive earlier after symptom onset to the hospital and are more likely to arrive in ambulance, compared to un-insured patients. Patients with insurance are also more likely to be treated in designated primary stroke centers and more likely to be discharged to inpatient rehab facility. No difference was found however in length of stay in the acute hospital, in prescription of drugs, in stroke education given, or in any of the stroke outcome measures: the severity of disease at arrival (measured with the NIH Stroke Scale) or disability level at discharge (measured with the modified Rankin Scale) by insurance status. In Belgium, all patients are mandatorily insured through the tax-funded, national health care system, and in addition to that an estimated 80% have a complimentary hospitalization insurance covering the difference between the actual hospitalization costs and what is reimbursed by the compulsory insurance. These insurances are contracted in 50% of cases by employers, in 33% taken by patients as an additional insurance with their sickness fund and in 17% of cases patients pay for an individual health insurance plan [22, 23]. Future research could focus on whether the resources found in this study differ within each SOI category by insurance status; unfortunately this data was not available in our dataset.

DRG-based funding for hospital admissions thus performs best when the variability in resources used by patients during a hospital admission is low, because costs are predictable and the financial risk can be managed equitably between the provider and the payer. The Belgian Health Care Knowledge Centre (KCE) published a study to investigate which APR DRG-SOI have low variability and are particularly suited to have full DRG funding [24]. In APR-DRG 045 ischaemic stroke, two SOIs were found to have very predictable resource utilisation: the least frequent category SOI 1 (5%), and the most frequent category SOI 2 (53%). For those SOI levels it was found that most hospitals have procedures on how to manage these patients, and moreover that these procedures do not vary much between hospitals, resulting in low variability within and between hospitals in reimbursement level and in LOS data. However, for higher SOI levels 3 and 4 within APR-DRG 045 ischaemic stroke, the variability within a hospital and between hospitals was found to be considerably larger, indicating that patient characteristics and comorbidities vary more widely and there are also more differences between hospitals in how much resources are used to manage these patients. In our dataset, the variability in LOS in the SOI 1 and 2 was markedly lower than in the higher SOI classes, confirming these findings. As the latter patient groups are less homogenous in their used resources, fully funding these patients on the basis of a diagnosis-related categorisation is more challenging. Our data also confirmed the finding that patients in higher SOI levels have more comorbidities per person and have comorbidities affecting different organ systems resulting in a higher amount of medical resource utilisation. Differentiating the APR-DRGs by severity is a necessity in order to determine the appropriate level of funding for the hospitals who are managing these patients.

### The use of stroke-related diagnosis codes

Two results emerging from the ICD-9 code analysis merit highlighting.

Firstly, nearly two thirds of all ischaemic stroke hospitalisations were assigned a primary diagnosis code that was not event-specific (ICD-9 434.91, 64.5%). The use of more specific ICD-9 codes on the type of event (thrombosis or embolism of the cerebral arteries; or occlusion and stenosis of the precerebral arteries) as primary diagnosis was associated with higher SOI thus higher funding. More severe SOI categories were also found to be related to higher use of diagnostic imaging tests in our first analysis, which could explain the ability of the hospital to be more precise on the localisation of the ischaemia in the brain. However, higher SOI could not be individually associated with one of the more specific stroke codes.

Secondly, we observed frequent use of stroke-related ICD-9 codes as secondary diagnoses in our dataset (24%). This indicated that the same stroke was coded multiple times in the medical file within the same episode of care. This multiple stroke coding could be an indication of another ischaemia found in another location of the brain, in addition to the first event mentioned in the primary diagnosis code, which is clinically possible but not as frequently as found in this dataset. Another explanation for this multiple stroke coding is the fact that in the APR-DRG system every speciality/ward type in the hospital must assign a primary diagnosis code when a patient is admitted, and that the final DRG code of the hospitalisation is assigned by a coding specialist after discharge of the patient, based on the combined primary and secondary codes from each speciality/ward type in the medical file. Stroke patients typically enter the hospital through the Accident & Emergency (A&E) department (first speciality), followed by an admission in a neurological ward (second speciality). Some patients move on to a rehabilitation ward (third speciality), resulting in three primary diagnosis codes that are not necessarily identical. The A&E department is more likely to assign a non-specific stroke code at admission, whereas the neurology department will assign an event-specific or location-specific stroke ICD-9 code following imaging tests. The rehabilitation ward may focus more on neurological deficits as primary reason for admission into the ward. Only one of these primary diagnosis codes will be retained as the DRG-determining code (that will also determine the funding for the hospital), whereas the other codes may be retained as secondary diagnosis codes (determining the SOI). The 3M coding algorithm allows for these ward-specific coding practice and should correct for the double-counting of stroke codes. The final explanation of the multiple coding of stroke is the upcoding of a patient’s medical file in order to enhance funding for the hospital. However, given the complexity of the ward-specific coding, the imprecision of the ICD-9 coding system regarding ischaemic stroke (allowing to specify the location of the ischaemia in the precerebral arteries but not in the cerebral arteries) and the proprietary nature of the 3M algorithm, this is difficult to disentangle.

### Secondary codes and their relationship with the funding level

Comorbidities occurring frequently among stroke patients and leading to a higher funding level are atrial fibrillation and diabetes; whereas other risk factors such as a history of stroke and/or myocardial infarction, atherosclerosis, hypertension, hypercholesterolaemia,... to which neurologists usually take into account when making their treatment decisions did not automatically lead to higher funding for the hospital. A list of other comorbidities and complications were identified that were significantly more related to SOI levels of 3 and 4, including lung infections, renal insufficiency, end-of-life status and others. Not surprisingly, we found that the total number of comorbidities entered in the patient’s file determine the severity level of the admission. These findings demonstrate the importance for hospitals of collecting exhaustive information on pre-existing comorbidities, and updating the patient’s medical file when additional clinical information becomes available on any complications or comorbidities. But equally, from the view point of the National Health System, it is important to create sufficient quality checks on the coding to avoid misuse of the system by double-coding or up-coding admissions to a higher severity level in order to increase funding. Ensuring high-quality data collection and investing in medical coding is a necessity in order to attribute the correct amount of funding for the case mix of patients. This raises interesting questions and challenges regarding the governance set-up of DRG systems, turning the spotlight on the need for accurate data collection and coding if the key principle of DRG systems is to be achieved: to balance the financial risk between the health care provider and the payer.

### Limitations of the study

We used two different datasets to draw our conclusions, and both have limitations. The first dataset with resource use data was based on amounts of use for each type of resource, per year and per hospital. This made it difficult to calculate confidence intervals for total costs; as no correlation data was available between the many different types of resources within each category. We were able to report the variability around LOS, and this variable was the most important component of total costs.

Another caveat should be entered, as the selection process for creating our second dataset on primary and secondary ICD-9 codes by APR-DRG/SOI was based on the primary diagnosis group being APR-DRG "045 Ischaemic stroke", which could be a limitation. A study by Tirschwell and colleagues demonstrated that reported stroke outcomes depended on the administrative selection process of the records [25]. The authors found that excluding patients with an ischaemic stroke diagnosis in a non-primary position resulted in underestimation of the hospitalisation cost. Furthermore, this analysis was performed in a single academic centre. Statistically significant findings were recorded, but the results from this study need to be replicated and, ideally, expanded upon using larger study samples.

We also used data based on the ICD-9 coding of medical information; many countries including Belgium have recently moved onto ICD-10 coding but national data based on ICD-10 will not be available for a number of years (in Belgium this is expected from 2018 onwards). Mapping functions exist between ICD-9 and ICD-10 [26], but are not always 1-on-1, therefore some ICD-9 codes have more than one corresponding code in the ICD-10 classification, hence the difficulty of transposing our results onto the newer system. Using this mapping function, the ICD-9 code 434.91 “Unspecified occlusion of the cerebral artery, with mention of brain infarct” that was most frequently used as primary diagnosis code in our dataset, but has the disadvantage of being non-specific regarding the type of event and its location in the cerebral arteries, can be mapped onto the unique ICD-10 code I63.50 “Cerebral infarction due to unspecified occlusion or stenosis of unspecified cerebral artery”. This confirms the imprecision of this stroke diagnosis code: it is imprecise about the type of event, as well as its location in the territory of the cerebral arteries. These arteries can be technically subcategorised into the following locations: anterior cerebral artery, middle cerebral artery, posterior cerebral artery (each of those split up between right, left, or bilateral location), cerebellar artery, other cerebral artery or “unspecified” cerebral artery. The new coding provides a different ICD-code for each event type (thrombosis or embolism) and for each of these locations. ICD-10 is therefore much more precise in the location of the ischaemia, which will result in more accurate, location-specific and event-type specific coding of the stroke, which in turn will lead to better attribution of funding.

### Policy implications

Creating severity subcategories of the APR-DRG “045 Ischaemic stroke”, which are defined by the number of organ systems affected rather than by the severity of the stroke itself, is a useful classification system that will cluster stroke patients into more homogenous groups in terms of resource use, and thus lead to a more efficient funding of these hospital admissions. Many countries using DRG-classifications as a funding model try to break down patients into smaller, more uniform clusters, either by creating subdivisions of the DRG, such as the SOI discussed in this study (used in 30 states in the US, Belgium, Spain, Italy, and in some Arab countries); or by creating a higher number of base DRGs (for example Germany creating 5000 base DRGs, the Dutch DOT system has 3000 DRGs); or by associating complication levels (no, minor, major complications) to the main DRG (such as done in US states using MS-DRG, in France with GHM, in Portugal with AP-DRG). Of all these systems, the APR-DRG with SOI is the most refined and advanced, collecting data on the principal diagnosis, age, interactions of multiple secondary diagnoses, and combinations of non-operating procedures with principal diagnosis, which is not available in these other systems. Moreover, its data structure and coding methodology enables to easily retrieve patients with specific conditions across different DRGs, which is particularly suitable for “population management”, i.e. preventive medicine in targeted populations. The identification of patients with particular chronic conditions for example is not possible or more difficult in the other systems, mainly due to their more limited data collection but also due to their data structure not allowing to query across different DRGs.

## Conclusion

In conclusion, an efficient funding system for ischaemic stroke hospitalisations should be based on clustering patients with similar needs of medical resources. The APR-DRG system with SOI subcategories classifies patients according to their primary (stroke) diagnosis plus a wide range of secondary diagnoses into four severity levels. Each severity level requires a different mix of resources necessary to manage the patient’s stroke plus any other affected organ systems, and will therefore lead to a different need of funding. Selected comorbidities were found to be associated with higher SOI, and hence higher funding received by hospitals. Ensuring high-quality data collection and investing in medical coding is a necessity to attribute the correct amount of funding for the case mix of patients.

The detailed data on medical care utilisation reported by severity level can be used in conjunction with unit costs from other countries with similar healthcare set-ups to 1) assess stroke-related hospital funding versus actual costs; 2) estimate the cost-effectiveness and budget impact of stroke prevention measures and treatment. The data on diagnosis codes and their link with SOI can be used to 3) understand which factors apart from stroke severity can influence the funding received by hospitals; 4) raise awareness about medical coding practices and double-coding coding to influence hospital funding.

## Additional files

Additional file 1: **Table S1.** “Overview of laboratory tests and cost calculation”: this file contains a data table detailing typical lab tests carried out among stroke patients and the calclation of total cost. (DOCX 49 kb)

Additional file 2: **Table S2.** “Overview of medical imaging procedures and cost calculation per SOI”: this file contains a data table with the rate of use of 49 different imaging tests, per SOI, and calculations of total imaging costs. (DOCX 54 kb)

## Abbreviations

A&E: Accident & Emergency
AP-DRG: All-Patient Diagnosis-Related Group
APR-DRG: All-Patient Refined Diagnosis-Related Groups
CVA: Cerebrovascular Accident or Stroke
DNR: Do Not Resuscitate
DOT: Dutch funding system On the way to Transparency using Diagnosis-Treatment Combinations
DRG: Diagnosis-Related Groups
GHM: Groupes Homogènes de Malades
ICD: International Classification of Diseases
KCE: Belgian Health Care Knowledge Center
LOS: Length of Stay
MS-DRG: Medicare Severity Diagnosis-Related Group
NIHDI: National Institute for Health and Disability Insurance
NIHSS: National Institute of Health Stroke Scale
SAS: Statistical Analysis System
SD: Standard Deviation
SOI: Severity of Illness
TCT: Belgian national hospitalizations database created by the Technical Cell
UTI: Urinary Tract Infection

## References

[1] Mozaffarian D, Benjamin EJ, Go AS, Arnett DK, Blaha MJ, Cushman M (2015). Heart disease and stroke statistics--2015 update: a report from the American Heart Association. Circulation.

[2] Carod-Artal FJ, Egido-Navarro JA, Gonzalez-Gutierrez JL, Varela de Seijas E (1999). Direct cost of cerebrovascular disease during the first year of follow-up. Rev Neurol.

[3] Spieler JF, Lanoe JL, Amarenco P (2002). Socioeconomic aspects of postacute care for patients with brain infarction in France. Cerebrovasc Dis.

[4] Andlin-Sobocki P, Jonsson B, Wittchen HU, Olesen J (2005). Cost of disorders of the brain in Europe. Eur J Neurol.

[5] Ekman M (2004). Economic evidence in stroke: a review. Eur J Health Econ.

[6] Truelsen T, Ekman M, Boysen G (2005). Cost of stroke in Europe. Eur J Neurol.

[7] Moon L (2003). Stroke care in OECD countries: a comparison of treatment, costs and outcomes in 17 countries, OECD health working papers.

[8] Schreyogg J, Stargardt T, Tiemann O, Busse R (2006). Methods to determine reimbursement rates for diagnosis related groups (DRG): a comparison of nine European countries. Health Care Management Sci.

[9] O'Reilly J, Busse R, Hakkinen U, Or Z, Street A, Wiley M (2012). Paying for hospital care: the experience with implementing activity-based funding in five European countries. Health Economics Policy Law.

[10] Turner-Stokes L, Sutch S, Dredge R, Eagar K (2012). International casemix and funding models: lessons for rehabilitation. Clinical Rehabil.

[11] 3M Statement – M. Sivertsen, International Marketing Manager 3M HIS.

[12] All Patient Refined DRGs (APR DRGs) Methodology Overview. 3M Health Information Systems. Consulting Services©. 2006.

[13] Cleemput I, Neyt M, Van de Sande S, Thiry N. Belgian guidelines for economic evaluations and budget impact analyses: second edition. Health Technology Assessment (HTA). Brussels: Belgian Health Care Knowledge Centre(KCE). 2012. KCE Report 183C. D/2012/10.273/54.

[14] Nomensoft. Nomenclatuur van de geneeskundige verstrekkingen. http://ondpanon.riziv.fgov.be/Nomen/nl/search. Accessed 15 July 2017.

[15] Technische cel voor de verwerking van de gegevens met betrekking tot de ziekenhuizen/cellule technique de traitement de données relatives aux hôpitaux 2014. https://tct.fgov.be/webetct/etct-web/html/nl/index.jsp. Accessed July 2017.

[16] Tsang TS, Petty GW, Barnes ME, O'Fallon WM, Bailey KR, Wiebers DO (2003). The prevalence of atrial fibrillation in incident stroke cases and matched population controls in Rochester, Minnesota: changes over three decades. J Am College Cardiol.

[17] Goldstein LB, Samsa GP, Matchar DB, Horner RD (2004). Charlson index comorbidity adjustment for ischemic stroke outcome studies. Stroke.

[18] Fischer U, Arnold M, Nedeltchev K, Schoenenberger RA, Kappeler L, Hollinger P, et al. Impact of comorbidity on ischemic stroke outcome. Acta Neurol Scand. 2006;113(2):108–13. 10.1111/j.1600-0404.2005.00551.x 16411971

[19] Karatepe AG, Gunaydin R, Kaya T, Turkmen G (2008). Comorbidity in patients after stroke: impact on functional outcome. J Rehabil Med.

[20] Henry J Kaiser Family Foundation. Health Insurance Coverage of the Total Population. 2016. http://kff.org/state-category/health-coverage-uninsured/health-insurance-status. Accessed 23 Jan 2018.

[21] Medford-Davis LN, Fonarow GC, Bhatt DL, Xu H, Smith EE, Suter R, et al. Impact of insurance status on outcomes and use of rehabilitation Services in Acute Ischemic Stroke: findings from get with the guidelines-stroke. J Am Heart Assoc. 2016;5(11):e004282. 10.1161/JAHA.116.004282 PMC5210352 27930356

[22] Assuralia, Assurinfo, National Spending on Health Care (Nationale Uitgaven in de Gezondheidszorg), 2017 (13), pp21. http://www.assuralia.be/images/docs/stats/NL/01_Assuraliastudies/nationale-gezondheidsuitgaven-cijfers2014.pdf. Accessed 23 Jan 2018.

[23] Trends. Bijna 8 miljoen Belgen hebben hospitalisatieverzekering. http://trends.knack.be/economie/finance/bijna-8-miljoen-belgen-hebben-hospitalisatieverzekering/article-normal-223509.html. Accessed 23 Jan 2018.

[24] Devriese S, Van de Voorde C. Clusteren van pathologiegroepen volgens gelijkenissen tussen ziekenhuisverblijven – Synthese. Health Services Research (HSR). Brussel: Federaal Kenniscentrum voor de Gezondheidszorg (KCE). 2016. KCE Reports 270A. D/2016/10.273/60.

[25] Tirschwell DL, Longstreth WT (2002). Validating administrative data in stroke research. Stroke.

[26] ICD10Data.com. http://www.icd10data.com/. Accessed 15 July 2017.

