# Supplementary material for: Hospital financing of ischaemic stroke: determinants of funding and usefulness of DRG subcategories based on severity of illness
Source: BMC Health Serv Res. 2018 May 11;18:356. doi: 10.1186/s12913-018-3134-6 (PMC5946535; doi:10.1186/s12913-018-3134-6)
Supplement: Supplementary file 1 — Table S1. “Overview of laboratory tests and cost calculation”: this file contains a data table detailing typical lab tests carried out among stroke patients and the calclation of total cost. (DOCX 49 kb) [file 12913_2018_3134_MOESM1_ESM.docx]

**Title:** Hospital financing of ischaemic stroke: determinants of funding and usefulness of DRG subcategories based on Severity of Illness

**Authors:** Sarah Dewilde^1,2^, Lieven Annemans^2,3^, Hilde Pincé^4,5^, Vincent Thijs^6^

**Document:** Online Resource Table 1

**Affiliations:**

1 Department of Public Health, Faculty of Medicine, UGent, Belgium

2 Services in Health Economics, Brussels, Belgium

3 Interuniversity Centre for Health Economics Research UGent, VUB, Belgium

4 UZ Leuven, Belgium

5 KU Leuven Institute for Healthcare Policy, Belgium

6 Florey Institute of Neuroscience and Mental Health, University of Melbourne and Austin Health, Department of Neurology, Heidelberg, Victoria, Australia.

**Online Resource Table 1: Overview of laboratory tests and cost calculation**

| **Test** | **Test description** | **Unit cost** |
| --- | --- | --- |
| Haematology | Measuring of the plasma viscosity | €0.57 |
| Coagulation | Measuring of the activated coagulation time | €2.04 |
| Renal function | Dosing of creatinine | €0.57 |
| Liver function | Dosing of albumin | €0.65 |
|  | Dosing of total bilirubin and its fractions | €0.65 |
|  | Thromboplastin time (prothrombin time), including calculation of fibrinogen | €0.65 |
| Electrolytes | Dosing of sodium | €0.49 |
|  | Dosing of calcium | €0.49 |
| Heart enzymes | Electrophoresis with diagram of the calculation of isoenzymes of creatine kinase | €1.22 |
| Glucose | Dosing of glucose or other reducing sugars | €0.49 |
| Lipid profile | Dosing of total cholesterol | €0.57 |
|  | Dosing of HDL-cholesterol | €0.82 |
|  | Dosing of LDL-cholesterol, excluding calculation methods | €0.82 |
|  | Dosing of triglycerides | €0.57 |
| CRP | Dosing of CRP with an immunological method | €0.82 |
| Total for fee-for-service cost | | €11.42 |
| Multiplication factor of 5 to account for lump sum payments from Sickness Funds to hospitals ^a^ | | €57.10 |
| Additional funding through patient share per in-patient stay | | €7.44 |
| **Total cost for laboratory tests** | | **€64.54** |

CRP: C-reactive protein; HDL: high-density lipoprotein; LDL: low-density lipoprotein

^a^ Costing method recommended by the Belgian Health Care Knowledge Centre: the multiplication factor converts the fee-for-service-based funding into total funding by accounting for the lump sum payments received from the national health care system
