# Supplementary material for: Hospital financing of ischaemic stroke: determinants of funding and usefulness of DRG subcategories based on severity of illness
Source: BMC Health Serv Res. 2018 May 11;18:356. doi: 10.1186/s12913-018-3134-6 (PMC5946535; doi:10.1186/s12913-018-3134-6)
Supplement: Supplementary file 2 — Table S2. “Overview of medical imaging procedures and cost calculation per SOI”: this file contains a data table with the rate of use of 49 different imaging tests, per SOI, and calculations of total imaging costs. (DOCX 54 kb) [file 12913_2018_3134_MOESM2_ESM.docx]

**Title:** Hospital financing of ischaemic stroke: determinants of funding and usefulness of DRG subcategories based on Severity of Illness

**Authors:** Sarah Dewilde^1,2^, Lieven Annemans^2,3^, Hilde Pincé^4,5^, Vincent Thijs^6^

**Document:** Online Resource Table 2

**Affiliations:**

1 Department of Public Health, Faculty of Medicine, UGent, Belgium

2 Services in Health Economics, Brussels, Belgium

3 Interuniversity Centre for Health Economics Research UGent, VUB, Belgium

4 UZ Leuven, Belgium

5 KU Leuven Institute for Healthcare Policy, Belgium

6 Florey Institute of Neuroscience and Mental Health, University of Melbourne and Austin Health, Department of Neurology, Heidelberg, Victoria, Australia.

**Online Resource Table 2: Overview of medical imaging procedures and cost calculation by SOI (bottom-up cost)**

| **Test description** | **Fee for service** | **SOI 1** | **SOI 2** | **SOI 3** | **SOI 4** | **ALL** |
| --- | --- | --- | --- | --- | --- | --- |
|  |  |  |  |  |  |  |
| Limited transthoracic echographic workup of the heart, whereby two-dimensional images are obtained, and Doppler signals in spectral mode. The recording and archiving of the examination is required, as well as a brief description of the clinical problem. | €36.33 | 0.141 | 0.327 | 0.340 | 0.357 | 0.331 |
| Complete transthoracic echographic workup of the heart, whereby two-dimensional images are obtained in at least three different cross-sections, and color-Doppler signals and in spectral mode at the height of at least three valve openings. The recording and archiving of the examination on magnetic tape or on digital carrier is required, as well as a detailed protocol. | €63.21 | 0.313 | 0.221 | 0.158 | 0.123 | 0.190 |
| Complete transesophageal echographic workup of the heart, whereby two-dimensional images are obtained in at least three different cross-sections, and color-Doppler signals at the height of at least three valve openings. The recording and archiving of the examination on magnetic tape or on digital carrier is required as well as a detailed protocol. | €115.59 | 0.172 | 0.141 | 0.124 | 0.133 | 0.136 |
| Fibroduodenoscopy (2nd and 3rd duodenum) | €125.54 | 0.016 | 0.042 | 0.051 | 0.075 | 0.049 |
| Evoked brain potentials with protocol and route extracts - by visual stimulation: - by auditive stimulation: - by somesthetic stimulation, including possible measurements carried out on spinal level of the plexus brachialis or the peripheral nerves (should not be cumulated with the electromyography): one of the above mentioned examinations. | €86.84 | 0.047 | 0.054 | 0.041 | 0.032 | 0.046 |
| Electroencephalographic examination with report | €69.55 | 0.531 | 0.317 | 0.222 | 0.222 | 0.281 |
| Limited transesophageal echographic workup of the heart, whereby two-dimensional images are obtained, and color-Doppler signals in spectral mode. The recording and archiving of the examination is required, as well as a brief description that answers the clinical problem | €59.45 | 0.000 | 0.011 | 0.024 | 0.044 | 0.020 |
| Bilateral color duplex examination of the carotid artery | €62.09 | 0.000 | 0.004 | 0.000 | 0.003 | 0.002 |
| Bilateral color duplex examination of the carotid artery and of the vertebral artery | €73.98 | 0.397 | 0.185 | 0.098 | 0.058 | 0.145 |
| Bilateral color duplex examination of the carotid artery | €62.09 | 0.000 | 0.011 | 0.031 | 0.033 | 0.020 |
| Bilateral color duplex examination of the carotid artery arteria carotis and of the vertebral artery | €73.98 | 0.127 | 0.193 | 0.407 | 1917.000 | 0.545 |
| Bilateral color duplex examination with a complete workup of the superficial and deep venous blood vessels of both upper or both lower limbs | €52.84 | 0.175 | 0.318 | 0.603 | 1325.000 | 0.565 |
| Repetition within the calendar year of the provisions 469814 - 469825 or 460456 - 460460 for one of the following indications. The recording and archiving of the examination on magnetic tape or digital devices is required, as well as a detailed protocol and the record maintaining of the repeat examinations. | €63.21 | 0.016 | 0.016 | 0.015 | 0.025 | 0.017 |
| Rectosigmoidoscopy or coloscopy left | €47.55 | 0.143 | 0.184 | 0.205 | 0.146 | 0.182 |
| Full colonoscopy, i.e. till the right corner of the colon or the ileocecal valve | €167.16 | 0.000 | 0.003 | 0.002 | 0.003 | 0.002 |
| Electromyography | €74.90 | 0.016 | 0.005 | 0.005 | 0.003 | 0.005 |
| Measurement of the (motor and/or sensitive) nerve conduction velocity and/or myasthenic tests and/or Hoffman reflex and/or F waves, one or more sites, including report and at least two tests. | €47.55 | 0.016 | 0.012 | 0.022 | 0.019 | 0.016 |
| Exploration of the integrity and the conduction velocity of the central motor fibers by percutaneous magnetic stimulation of the motor cortex | €81.27 | 0.000 | 0.010 | 0.005 | 0.011 | 0.008 |
| Holter Monitoring: continuous electrocardiographic registration for at least 24 hours, by means of a portable device with magnetic tape or with built-in memory, including consultation when installing and removing the device, with protocol and possibility to reproduce the entire routes. | €76.09 | 0.270 | 0.144 | 0.070 | 0.055 | 0.112 |
| Repeat within one year provisions nr 476210 - 476221 | €57.07 | 0.016 | 0.011 | 0.000 | 0.008 | 0.008 |
| Evoked brain potentials with protocol and route extracts - by visual stimulation; - by auditive stimulation; - by somesthetic stimulation, including possible measurements carried out on spinal level of the plexus brachialis or the peripheral nerves (should not be cumulated with the electromyography): two examinations | €133.15 | 0.016 | 0.007 | 0.024 | 0.011 | 0.013 |
| Evoked brain potentials with protocol and route extracts - by visual stimulation; - by auditive stimulation; - by somesthetic stimulation, including possible measurements carried out on spinal level of the plexus brachialis or the peripheral nerves (should not be cumulated with the electromyography): the three examinations | €173.67 | 0.000 | 0.005 | 0.008 | 0.006 | 0.006 |
| Nuclear magnetic resonance examination of the head (skull, brains, petrous bone, pituitary, sinuses, orbital(e) or maxillary joints, at least three sequences, with our without contrast, with recording on optical or electromagnetic carrier | €93.23 | 0.406 | 0.303 | 0.180 | 0.115 | 0.240 |
| Radiography of the swallowing mechanism pharynx-hypopharynx, with radioscopic examination with image intensifier and television in closed chain, minimum of six clichés | €42.72 | 0.000 | 0.039 | 0.041 | 0.018 | 0.035 |
| Radiography of the thorax and its content, one cliché | €12.57 | 0.094 | 0.097 | 0.222 | 0.489 | 0.197 |
| Radiography of the thorax and its content, minimum two clichés | €15.08 | 0.206 | 0.127 | 0.090 | 0.085 | 0.112 |
| Computed tomography of the skull with or without contrast agent, including registration and clichés, minimum 10 cuts for the entire examination | €92.31 | 0.453 | 0.373 | 0.372 | 0.463 | 0.390 |
| Computed tomography of the neck (soft tissues) with or without contrast agent, including registration and clichés, minimum 15 cuts for the entire examination. | €133.33 | 0.281 | 0.372 | 0.390 | 0.410 | 0.379 |
| Additional fee for subtraction method in cerebral or visceral angiography, including the mask and the positive: maximum for the entire examination | €27.88 | 0.000 | 0.001 | 0.000 | 0.000 | 0.000 |
| Isolated vertebral cerebral angiography, minimum two different angles and 10 clichés | €139.40 | 0.000 | 0.363 | 0.355 | 0.234 | 0.346 |
| Magnetic resonance angiography of the cervical vessels or of the thoracic or of the abdominal or of the pelvic blood vessels or of a limb, at least three sequences, with or without contrast agent, including registration on an optical or electromagnetic carrier | €178.79 | 0.031 | 0.038 | 0.023 | 0.018 | 0.030 |
| Holter Monitoring: continuous electrocardiographic registration for at least 24 hours, by means of a portable device with magnetic tape or with built-in memory, including consultation when installing and removing the device, with protocol and possibility to reproduce a part of the routes | €47.55 | 0.000 | 0.000 | 0.003 | 0.000 | 0.001 |
| Measuring of the motor and/or sensitive conductor velocity, different sites, with report | €35.76 | 0.000 | 0.000 | 0.007 | 0.006 | 0.003 |
| By auditive stimulation evoked brain potentials and/or electrocochleographic examination with protocol and route extract | €93.50 | 0.037 | 0.092 | 0.194 | 0.229 | 0.147 |
| Preoperative digital arteriography of the carotid artery | €33.92 | 0.259 | 0.521 | 0.552 | 0.564 | 0.533 |
| Computed tomography of the abdomen, with or without contrast agent, including registration and clichés, minimum 15 cuts, for the entire examination | €133.33 | 0.556 | 0.382 | 0.516 | 0.662 | 0.478 |
| Color duplex examination of the intracranial vessels | €54.82 | 1.370 | 1.026 | 1.304 | 1.909 | 1.280 |
| Fibrogastroscopy and/or fibrobulboscopy | €104.62 | 0.556 | 0.534 | 0.475 | 0.433 | 0.498 |
| Total amount of fee-for-service received by hospitals per patient per stroke admission |  | €515.63 | €509.58 | €547.10 | €733.80 | €562.83 |
| Multiplication factor of 1.7 to take into account lump sum payments received by hospitals from the Sickness Funds ^a^ |  | €876.57 | €866.29 | €930.07 | €1,247.46 | €956.81 |
| Additional funding received by hospitals through patient share per inpatient stay |  | €6.20 | €6.20 | €6.20 | €6.20 | €6.20 |
| **Total amount per patient per stroke admission for imaging** |  | **€882.77** | **€872.49** | **€936.27** | **€1,253.66** | **€963.01** |

^a^ Costing method recommended by the Belgian Health Care Knowledge Centre: the multiplication factor converts the fee-for-service-based funding into total funding by accounting for the lump sum payments received from the national health care system
